# Gastric Superacidity Treated by Cholic Acid

**Published:** 1914-08

**Authors:** 


**Gastric Superacidity Treated by Cholic Acid.** Glaessner, Wien. Klin. Woch., states that bile, by its salts of the cholic acids, inhibits the secretion of HCI and pepsin. He uses cholic acid in doses of 5-6 centigrams daily.

